# Ecosystem restoration, regeneration and rewilding

**DOI:** 10.1186/s12862-023-02165-3

**Published:** 2023-09-14

**Authors:** Nancy Shackelford, Carmel McDougall

**Affiliations:** 1 Nancy Shackelford, School of Environmental Studies, University of Victoria, Victoria, Canada; 2 Carmel McDougall. Scottish Oceans Institute, University of St Andrews, St Andrews, Scotland

## Abstract

Anthropomorphic activities have caused major damage to ecosystems worldwide. Although documenting this damage is important, implementing measures to halt and reverse ecosystem decline is critical and is now being prioritised globally. To support global goals to protect and restore nature, *BMC Ecology and Evolution* has launched a new article collection to encourage contributions from the multifaceted ecosystem restoration community.

## Main text

The world is turning to restoration as a means of addressing many of the environmental issues that we face globally. The United Nations (UN) General Assembly declared 2021–2030 as the UN Decade on Ecosystem Restoration. In December 2022, world leaders adopted the Kunming-Montreal Global Biodiversity Framework. The Framework outlines goals for 2050 to be achieved via quantitative targets actioned by 2030 to protect and restore nature [1]. Restoration can help fight climate change through carbon sequestration, buffer coastlines from rising sea levels, prevent species extinctions, mitigate the impacts of resource extraction and modern consumption practices, protect and restore the provision of ecosystem services, reconnect people with nature, and support Indigenous reconciliation movements. Restoration projects can differ widely in their objectives and involve diverse groups, including interdisciplinary scientists, practitioners, policymakers and local and Indigenous communities.

Various definitions of ‘restoration’ reflect the multifaceted field of restoration ecology. The Society of Ecological Restoration has had a steady definition since 2004 [2] – “the process of assisting the recovery of an ecosystem that has been degraded, damaged, or destroyed”. The UN Decade on Ecosystem Restoration defines it as “the process of halting and reversing degradation, resulting in improved ecosystem services and recovered biodiversity” [3]. The IUCN, in the context of agreements like the Bonn Challenge, specifically focuses on forest landscape restoration and defines it as “the ongoing process of regaining ecological functionality and enhancing human well-being across deforested or degraded forest landscapes” [4]. Ultimately, definitions of “restoration” differ broadly, and the word “restoration” has come to encompass a wide range of nature-based practices.

At its heart, however, is the idea that we as humans can assist the **regeneration** of nature – we can help reverse damage while perhaps also prioritising the return of the many declining services nature provides humanity. Historically, regeneration has focused on natural processes, either ecosystem recovery without intervention or recovery after deliberate removal of stressors, referred to as “assisted regeneration” or “passive restoration”. The term has, predictably, evolved over the last few decades. Just ten years ago, Higgs suggested it as a flexible alternative to the word restoration that would bring together the focus on ecological integrity with innovative techniques in nature-based engineering and design [5]. Thus, regeneration remains another term with a broad scope, narrowed only by its reliance on natural processes and nature’s power of recovery.

Another branch of restoration-related practice, ‘rewilding’, has experienced a surge in the last few decades. Originally a term used to describe protecting keystone species and their ecosystems by developing interconnected reserves, the meaning of ‘rewilding’ has now diversified and encapsulates “rebuilding, following major human disturbance, a natural ecosystem by restoring natural processes and the complete or near complete food web at all trophic levels as a self-sustaining and resilient ecosystem with biota that would have been present had the disturbance not occurred” [6]. Rewilding, like regeneration, focuses on natural processes as the template for intervention; in particular, leaning on trophic complexity, dispersal, and stochastic disturbance to lead to a self-sustaining and resilient “wild” ecological community [7], without prescribed endpoints for what that community might look like.

In this special Collection, we focus on these three terms - ecological restoration, regeneration, and rewilding (ERRR) - as terms that capture actions most clearly driven by ecological goals. Other terms, including ‘remediation’ or ‘rehabilitation’, are also on this spectrum but tend to be tied more closely with ecosystem services, functionality, and human-centred outcomes [8]. As the need and demand for nature-centred solutions continues to grow, it is essential that we advance our efficacy in these fields as a global community rather than in isolated silos. We continue to face key challenges in doing so, and hope the articles in this Special Collection will help progress us along the needed pathway of shared learning.

A major challenge for ERRR is the intrinsic multidisciplinary nature of the research. The importance of ecology for the successful implementation of ecosystem restoration is clear. However a range of other disciplines are also important. Economic analysis determines the viability and benefit of projects, and can also assess the value placed on healthy ecosystems by local residents and their ‘willingness to pay’ for proposed restoration activities (e.g., mangrove forest restoration in India and Bangladesh [9]). Genetic analysis is of growing importance to understand the remaining genetic diversity of populations, to determine the optimal source populations for species introductions, and for monitoring ongoing projects - both of the target species themselves, and also of general community biodiversity through environmental DNA analyses (e.g., assessment of biodiversity associated with artificial coral reefs [10]). Incorporation of engineering principles is also critical for many restoration projects, for example, the inclusion of breakwalls to alleviate boat wake disturbance to facilitate shellfish reef recovery [11]. These examples highlight just a few of the many disciplines that inform restoration research, and we invite submissions from any of these for publication in this Collection.

Restoration projects reconfigure relationships between people and nature in complex ways, influenced by factors such as project goals and leadership, decision-making scale, the level of community consultation, and the relationships between the stakeholders and partners. Inclusivity and diversity in ERRR projects can increase adaptive capacity in the face of rapid environmental change [12] and enhance the socio-ecological resilience of projects across space and time [13]. This needed diversity of voices should be reflected both in project implementation and in the scientific literature where expertise is shared. However, as seen in many scientific fields, certain voices in the ERRR literature need to be better represented. Those from Indigenous groups, non-Western societies and practitioner groups often have little incentive or support to publish.

For ERRR the engagement of diverse perspectives is likely more common in practice than in the literature. For example, shellfish reef restoration in the tropics is gaining momentum, yet most published research focuses on temperate regions. The lack of information from the tropics means that tropical oyster reefs have been excluded from global assessments [14]. Related issues can drive the exclusion of cultures and peoples from the scientific literature. Western researchers have dominated the literature around oak meadow restoration in British Columbia, yet these ecosystems evolved from Indigenous management, and most importantly they continue to be stewarded by local First Nations today [15]. The progress made through these project types is poorly documented in the global literature, and knowledge thus becomes siloed within the local participants. Though some knowledge may require privacy and should be respected, there are still shareable, important lessons to be learned from each other.

Thus, we aim to attract research from various disciplines and voices on the design, application, optimization, management, and outcomes of ERRR in terrestrial, freshwater, and marine ecosystems. We recognize that there may be barriers to publication for many but we welcome anyone interested in sharing their experiences through this collection to reach out to us, the collection editors, for support and assistance.

## Data Availability

Not applicable.

## Abbreviations

ERRR: Ecosystem restoration, regeneration and rewilding
IUCN: International Union for Conservation of Nature
UN: United Nations
